# Respiratory sarcopenia is a predictor of all‐cause mortality in community‐dwelling older adults—The Otassha Study

**DOI:** 10.1002/jcsm.13266

**Published:** 2023-06-14

**Authors:** Takeshi Kera, Hisashi Kawai, Manami Ejiri, Kumiko Ito, Hirohiko Hirano, Yoshinori Fujiwara, Kazushige Ihara, Shuichi Obuchi

**Affiliations:** ^1^ Department of Physical Therapy, Faculty of Health Care Takasaki University of Health and Welfare Takasaki Japan; ^2^ Research Team for Human Care, Tokyo Metropolitan Institute for Geriatrics and Gerontology Tokyo Japan; ^3^ Research Team for Promoting Independence and Mental Health Tokyo Metropolitan Institute for Geriatrics and Gerontology Tokyo Japan; ^4^ Tokyo Metropolitan Institute for Geriatrics and Gerontology Tokyo Japan; ^5^ Department of Social Medicine Hirosaki University School of Medicine Aomori Japan

**Keywords:** Community‐dwelling older adults, Forced vital capacity, Mortality, Peak expiratory flow rate, Respiratory sarcopenia, Respiratory muscle strength

## Introduction

As individuals age, skeletal muscle mass and function, including lean body mass and grip strength, and respiratory muscle mass and strength, tend to decline.^1^, ^2^ The term ‘respiratory sarcopenia’ emerged during a discussion on sarcopenia. Respiratory sarcopenia should encompass respiratory muscle mass and strength or function to adhere to the original sarcopenia definition, which considers whole‐body muscle mass, grip strength, and gait speed. However, the decreasing respiratory muscle mass associated with aging has not been adequately discussed. The concept may appear simple; however, defining respiratory sarcopenia has not been extensively explored.

Inspiratory and expiratory maximal mouth pressure measurement as direct evidence of respiratory muscle strength is simple; however, access to relevant measuring equipment is limited. Moreover, evaluating respiratory muscle mass is challenging, and the respiratory sarcopenia using low respiratory muscle mass cannot be virtually established. Therefore, we proposed defining respiratory sarcopenia using the peak expiratory flow rate (PEFR) as an alternative to directly measuring respiratory muscle strength.^3^ Subsequently, the Japanese Working Group of Respiratory Sarcopenia of the Japanese Association of Rehabilitation Nutrition (JARN) published criteria for respiratory sarcopenia, which was defined based on a decline in the maximal mouth pressure and respiratory muscle mass and the presence of whole‐body‐sarcopenia, as measured using skeletal muscle mass, strength, and physical performance.^4^ However, this definition has not been established due to a lack of consensus. Moreover, to the best of our knowledge, the future health‐related outcomes of respiratory sarcopenia have never been evaluated. Therefore, this survey confirmed whether respiratory sarcopenia, defined using PEFR and the JARN criteria, is associated with future mortality among community‐dwelling older adults.

## Methods

We assessed respiratory sarcopenia‐related mortality after a 5‐year follow‐up of 470 participants (185 men aged 75.2 ± 5.5 years and 285 women aged 74.2 ± 5.4 years) who participated in a comprehensive health checkup program called ‘The Otassha Study’ conducted in the Tokyo Metropolitan Institute for Geriatrics and Gerontology in 2015. Participants who underwent spirometry and sarcopenia assessment were included; however, patients with chronic obstructive pulmonary disease (COPD) were excluded.

An electronic spirometer (Autospiro AS‐507, Minato, Osaka, Japan) was used to measure pulmonary function. The PEFR as a percentage of the predicted value (%PEFR), vital capacity (VC), forced vital capacity (FVC), forced expiratory volume in 1 s (FEV_1_), VC as a percentage of the predicted value (%VC), FVC as a percentage of the predicted value (%FVC), lower limit of normal FVC (FVC_LLN_), and FEV_1_/FVC were assessed.

A multi‐frequency bio‐impedance body composition analyser (InBody 720, InBody. Co., Seoul, Korea) was used to measure skeletal muscle mass, and a manual stopwatch was used to determine gait speed along a 5‐m course with a 3‐m acceleration and deceleration area. Grip strength was measured using a Smedley‐type hand dynamometer while standing. Body mass index (BMI) was calculated as weight/height^2^.

Original sarcopenia was defined according to the criteria outlined by the Asian Working Group for Sarcopenia 2019.^5^ In the present study, respiratory sarcopenia was defined using the following two methods: (1) the PEFR, based on our previous study, and (2) the JARN criteria. In the first method, PEFR respiratory sarcopenia was defined when PEFR was lower than the cut‐off value (<4.40 L/s for men and <3.21 L/s for women).^3^ According to the JARN flowchart, respiratory sarcopenia is defined using respiratory muscle mass and maximal mouth pressure^4^; however, we did not measure respiratory muscle mass, which is difficult to measure, or maximal mouth pressure. Therefore, in the second method, respiratory sarcopenia, defined according to the JARN criteria, was diagnosed when the patient had both sarcopenia and low FVC. Participants were defined as having JARN respiratory sarcopenia when FVC was lower than FVC_LLN_ based on sex, age, and height. We defined both ‘definite’ and ‘probable’ respiratory sarcopenia as JARN respiratory sarcopenia.

## Results

Overall, 61 (13.0%) and 21 (4.5%) participants were classified as having PEFR and JARN respiratory sarcopenia, respectively, and 12 (2.6%) as having both.

During the 5‐year follow‐up, 31 of the 470 (6.6%) participants died. The 5‐year incidence of death was 8 (13.1%) and 5 (23.8%) for PEFR and JARN respiratory sarcopenia, respectively. The incidence of death for original sarcopenia was 13 (25.0%) (Table 1).

**Table 1:** Participant characteristics according to the presence of respiratory sarcopenia

Characteristics	Total (*n* = 470)	PEFR respiratory sarcopenia (*n* = 61; 13.0%)	JARN respiratory sarcopenia (*n* = 21; 4.5%)
Women, number (%)	285 (60.6)	34 (55.7)	13 (61.9)
Age, years	74.6 (5.5)	76.6 (6)	79.6 (5.7)
Height, cm	155.9 (8.3)	154.7 (8.7)	151.1 (7.9)
Weight, kg	55.4 (10.3)	53.7 (10)	49.7 (7)
Grip strength, kg	26.8 (7.8)	24.5 (8)	18.3 (5.7)
Walking speed, m/s	1.39 (0.25)	1.32 (0.27)	1.11 (0.29)
ASM/ht^2^, kg/m^2^	6.40 (0.97)	6.21 (1.03)	5.56 (0.76)
Lung function			
PEFR, L/s	5.39 (1.81)	2.91 (0.71)	3.35 (1.07)
FEV_1_, L	1.88 (0.5)	1.47 (0.43)	1.28 (0.34)
FEV_1_/FVC, %	79.6 (8.2)	74.9 (10.3)	77.8 (12.4)
VC, L	2.53 (0.71)	2.11 (0.52)	1.83 (0.42)
%VC (JRS), %	88.1 (15.6)	75.7 (13.6)	72.1 (12.6)
FVC, L	2.38 (0.64)	1.97 (0.56)	1.66 (0.46)
%FVC (JRS), %	86.2 (14.1)	73.4 (14.8)	67.1 (10.8)
Co‐morbidity			
Stroke, number (%)	35 (7.4)	5 (8.2)	1 (4.8)
Heart disease, number (%)	50 (10.6)	3 (4.9)	1 (4.8)
Diabetes, number (%)	62 (13.2)	10 (16.4)	18 (85.7)
COPD, number (%)	0 (0)	0 (0)	0 (0)
Original sarcopenia, number (%)	52 (11.1)	14 (23.0)	21 (100.0)
PEFR Respiratory sarcopenia, number (%)	61 (13.0)	‐	12 (57.1)
JARN Respiratory sarcopenia, number (%)	21 (4.5)	12 (19.7)	‐
5‐years death case, number (%)	31 (6.6)	8 (13.1%)	5 (23.8%)

Data are presented as mean (SD) and number (percentage).

%FVC (JRS), FVC % predicted using the Japan Respiratory Society method; %VC, VC % predicted using the Japan Respiratory Society method; ASM, appendicular skeletal muscle; COPD, chronic obstructive pulmonary disease; FEV_1,_ forced expiratory volume in 1 second; FVC, forced vital capacity; JARN, Japanese Association of Rehabilitation Nutrition; PEFR, peak expiratory flow rate.

**Figure 1:**
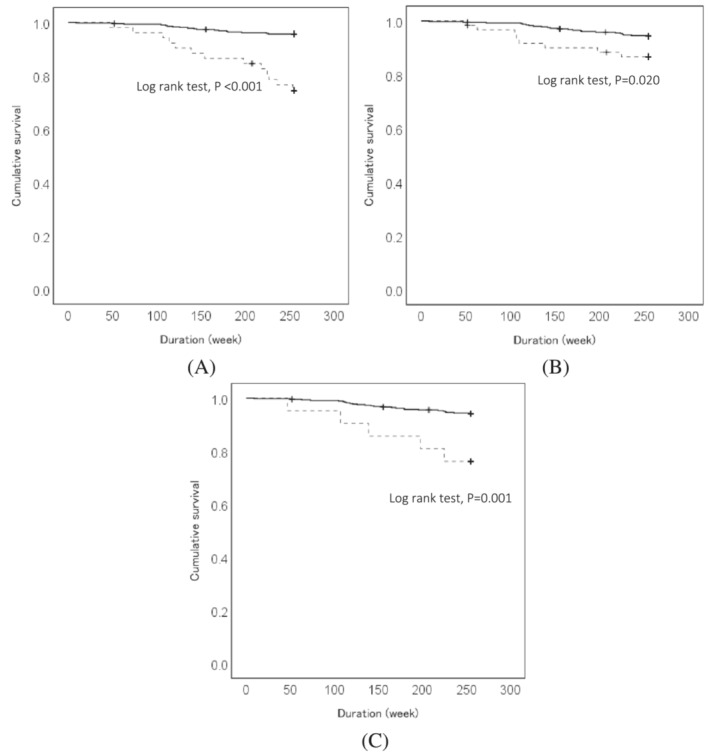
Kaplan–Meier survival curves exploring the association of cumulative survival with (A) original sarcopenia, (B) respiratory sarcopenia defined based on the PEFR, and (C) respiratory sarcopenia defined using the JARN criteria. JARN, Japanese Association of Rehabilitation Nutrition; PEFR, peak expiratory flow rate.

Figure 1 shows cumulative death results for original, JARN respiratory, and PEFR respiratory sarcopenia. Survival analysis using the Kaplan–Meier curve and log‐rank test showed that sarcopenia and PEFR and, JARN, respiratory sarcopenia were significantly associated with death during the 5‐year follow‐up. Patients with sarcopenia and PEFR and JARN respiratory sarcopenia had shorter survival durations than those without original and respiratory sarcopenia (log‐rank test, *P* < 0.001, *P* = 0.020, and *P* = 0.001, respectively).

Cox proportional hazard regression models were used to analyse the association of original, PEFR respiratory, and JARN respiratory sarcopenia with death (Table 2). In the crude model, patients with original sarcopenia (hazard ratio [HR], 6.36; 95% confidence interval [CI], 3.11–12.98; *P* < 0.001), PEFR respiratory sarcopenia (HR, 2.51; 95% CI, 1.12–5.62; *P* = 0.025), and JARN respiratory sarcopenia (HR, 4.52; 95% CI, 1.74–11.78; *P* = 0.002) at baseline had an increased mortality risk during the 5‐year follow‐up. Original and JARN respiratory sarcopenia was associated with increased mortality risk in model 2 (adjusted for BMI and comorbidity) and model 3 (adjusted for lifestyle and all variables in model 2). However, the HR of JARN respiratory sarcopenia (HR, 3.95; 95% CI, 1.50–10.39; *P* = 0.005) was close to that of original sarcopenia (HR, 3.05; 95% CI, 1.34–6.96; *P* = 0.008).

**Table 2:** Cox regression model of sarcopenia, JARN, and PEFR respiratory sarcopenia for 5‐year mortality

	Crude model		Model 2		Model 3	
	Hazard ratio (95% CI)	*P*	Hazard ratio (95% CI)	*P*	Hazard ratio (95% CI)	*P*
Original (Sarcopenia)	6.36 (3.11–12.98)	<0.001	3.27 (1.46–7.32)	0.004	3.05 (1.34–6.96)	0.008
Age			1.12 (1.05–1.20)	0.001	1.13 (1.05–1.21)	0.001
BMI			0.92 (0.81–1.04)	0.188	0.92 (0.81–1.04)	0.167
Cerebrovascular disease			1.30 (0.38–4.43)	0.678	1.26 (0.37–4.35)	0.711
Heart disease			0.77 (0.18–3.31)	0.724	0.79 (0.18–3.40)	0.748
Diabetes			1.27 (0.47–3.42)	0.641	1.01 (0.35–2.94)	0.980
Exercise					1.06 (0.82–1.36)	0.661
Smoking					0.73 (0.43–1.24)	0.249
PEFR (Respiratory sarcopenia)	2.51 (1.12–5.62)	0.025	1.61 (0.69–3.74)	0.268	1.54 (0.66–3.55)	0.315
Age			1.15 (1.08–1.23)	<0.001	1.16 (1.08–1.24)	<0.001
BMI			0.90 (0.80–1.01)	0.083	0.90 (0.80–1.01)	0.074
Cerebrovascular disease			1.06 (0.31–3.64)	0.924	1.03 (0.30–3.50)	0.968
Heart disease			0.63 (0.15–2.66)	0.526	0.65 (0.15–2.76)	0.556
Diabetes			1.28 (0.48–3.45)	0.624	1.06 (0.38–2.96)	0.912
Exercise					1.06 (0.83–1.36)	0.648
Smoking					0.67 (0.40–1.11)	0.121
JARN (Respiratory sarcopenia)	4.52 (1.74–11.78)	0.002	3.95 (1.50–10.38)	0.005	3.95 (1.50–10.39)	0.005
BMI			0.88 (0.77–0.99)	0.499	0.88 (0.78–1.00)	0.044
Cerebrovascular disease			1.53 (0.45–5.24)	0.475	1.40 (0.40–4.84)	0.595
Heart disease			0.59 (0.14–2.50)	0.575	0.60 (0.14–2.54)	0.488
Diabetes			1.33 (0.49–3.59)	0.005	1.16 (0.41–3.31)	0.779
Exercise					0.96 (0.76–1.23)	0.770
Smoking					0.69 (0.42–1.15)	0.156

95% CI, 95% confidence interval; BMI; body mass index; JARN, Japanese Association of Rehabilitation Nutrition; PEFR, peak expiratory flow rate; model 2, adjusted for BMI, stroke, heart disease, and diabetes; and model 3, model 2 + exercise and smoking.

**Figure 2:**
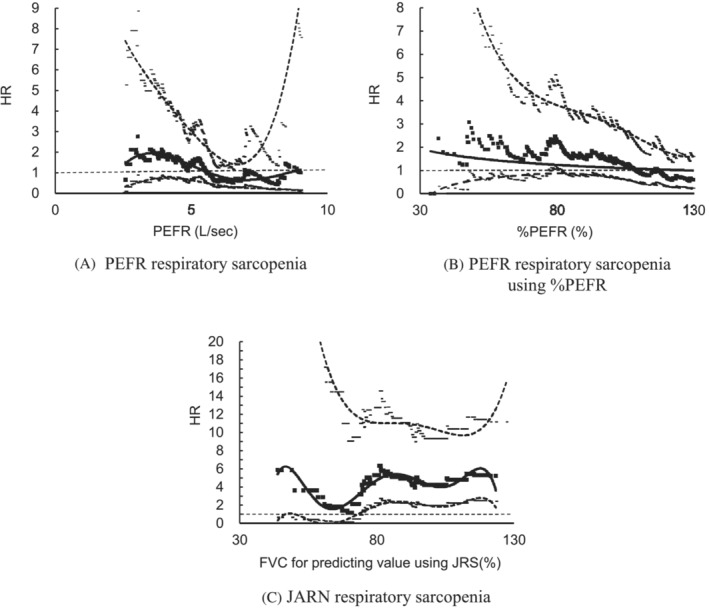
Association between change in each criterion for respiratory sarcopenia and mortality risk using model 3. The thick middle line represents the HR for mortality risk associated with each cut‐off value of the PEFR, %PEFR (PEFR for predictive value), and %FVC (FVC for the predictive value). Upper and lower thin lines represent the 95% CI of HR. Lines were drawn using a spline; the broken horizontal line represents an HR of 1.0. (A) The horizontal (*x*) axis is plotted based on the cut‐off value for men. The cut‐off value for women was set at 1.19 L/s, which was less than that for men. (A) HR for respiratory sarcopenia, defined using the PEFR, when its value was varied, (B) HR for respiratory sarcopenia when %PEFR was used instead of the PEFR, and (C) HR for respiratory sarcopenia, defined using the JARN criteria, when the %FVC cut‐off was varied. ■ and the solid line indicate, the HR; ‘‐’ and the dotted line indicate, lowest and highest 95% CI, respectively. CI, coefficient interval; HR, Hazard ratio; PEFR, peak expiratory flow rate; %PEFR, PEFR as a percentage of the predicted value; JARN, Japanese Association of Rehabilitation Nutrition; FVC, forced vital capacity; %FVC, FVC as a percentage of the predicted; JRS, Japanese Respiratory Association.

As the cut‐off values of FVC_LLN_ and PEFR proposed in this study and by JARN may be unsuitable for the consideration of future mortality, we varied the cut‐off values for %FVC, PEFR, and %PEFR, and observed the changes in the HR of mortality in model 3 (Figure 2). PEFR (Figure 2A) and %PEFR (Figure 2B), used for the definition of respiratory sarcopenia tended to have an HR value of >1.0 when the PEFR or %PEFR value was lower. The %PEFR cut‐off value of 76.1%–81.2% yielded a significant HR; however, the HR for the cut‐off value of respiratory sarcopenia using absolute PEFR (the value was 1.19 L/s lower in women than in men) was not significant among any of the PEFR values in model 3. With regard to the %FVC, the HR was significantly higher than 1.0; however, it was approximately constant between the low (73.6%) and high (127.0%) ranges of %FVC values (Figure 2C).

## Discussion

Cook et al. reported that community‐dwelling adults with low PEFR had higher mortality rates than those previously reported.^6^ This finding was also consistent with the findings that of Fragoso et al.^7^ Buchman et al.^8^ reported a relationship between mortality and some variables, including limb muscle strength, respiratory muscle strength, and lung function. Respiratory muscle strength was more strongly correlated with mortality than limb muscle strength. However, they might have included patients with COPD and other respiratory syndromes; therefore, this was a natural consequence. The HR for mortality obtained in the present study is similar to that in studies where the PEFR was used to define respiratory sarcopenia, even when COPD was excluded. However, this relationship was insignificant after adjusting for covariates. It is possible that the relationship between respiratory sarcopenia defined using PEFR and mortality was weak or that the cut‐off value for define PEFR was unsuitable. This implies that the cut‐off value recommended in our previous study, was unsuitable when mortality was set as a future health‐related outcome; therefore, a more suitable value is needed.

In contrast, the HR of JARN respiratory sarcopenia for mortality was significant, even after adjusting for covarites. Because this HR was close to that of original sarcopenia and the definition of JARN respiratory sarcopenia includes the concept of original sarcopenia, it may merely reflect sarcopenia. Mortality remained constant regardless of the fluctuating cut‐off values of %FVC, which were close to those of original sarcopenia. The relationship between JARN respiratory sarcopenia and 5‐year mortality appears to be strongly influenced by original sarcopenia. Accordingly, JARN respiratory sarcopenia may have only the same clinical implications as original sarcopenia. The HR of PEFR respiratory sarcopenia increased when low cut‐off values were set in the crude model; however, it was insignificant when adjusted for age. Furthermore, the HR for death was significant when using %PEFR cut‐off values between 76.1% and 82.6%. Therefore, we consider %PEFR a suitable indicator for predicting future health‐related outcomes among respiratory sarcopenia models.

Respiratory muscle mass and thickness are likely to change with age. However, the change in diaphragmatic thickness and echogenicity grayscale values is small.^9^ Therefore, respiratory muscle mass may not significantly reflect respiratory sarcopenia. However, maximal mouth pressure^10^ and PEFR,^11^ as measures of respiratory muscle function, correlate with whole skeletal muscle mass, physical function,^12^ and sarcopenia.^13^ Considering the conceptual definition of ‘Sarco,’ whole‐body or appendicular skeletal muscle mass might be useful in defining respiratory sarcopenia (rather than original sarcopenia) instead of respiratory muscle mass, which cannot be measured.

## Conflict of interest statement

None declared.

## Funding

The Otassha Study was supported by Research and Development Grants for Longevity Science from the Japan Agency for Medical Research and Development (AMED) (grant number: 15dk0107004h0003), Research grant from the National Center for Geriatrics and Gerontology (grant number: 20‐1), and JSPS KAKENHI (grant number: JP15K08824).
